# Can Antipsychotic Agents be Considered as Real Antimanic Treatments?

**DOI:** 10.3389/fpsyt.2014.00060

**Published:** 2014-05-26

**Authors:** Michel Bourin, Florence Thibaut

**Affiliations:** ^1^Neurobiologie de l’Anxiété et de la Dépression, Faculté de Médecine, University of Nantes, Nantes, France; ^2^Psychiatry and Addictive Disorders, University Hospital Cochin-Tarnier, INSERM 894 CPN, Faculty of Medicine Paris Descartes, Paris, France

**Keywords:** bipolar disorder, antipsychotic agents, mania, antiepileptics, antimanic agents

Until now, in various parts of the world, no consensus has been reached with regard to the treatment of acute mania. Controlled clinical trials have at last provided irrefutable evidence for the effectiveness of lithium, which had long been used alone, as well as that of divalproate or its derivatives and, to a lesser extent, carbamazepine (1). In Europe, haloperidol is still the reference compound used in clinical trials while it has never been officially approved in the treatment of mania. In the USA, lithium, divalproate, or second-generation-antipsychotics can be prescribed as first-line treatments. As dopamine was reported to be involved in the pathophysiology of mania in the 1970s and as changes in dopaminergic neurotransmission have consistently been reported in bipolar disorders (BDs), the question of the antimanic properties of antidopaminergic drugs such as antipsychotics is a fair one (2).

In Europe, lithium remains the first-line medication, whereas divalproate and atypical antipsychotic agents are mainly used as second-line treatments. Yet, manic inpatients are frequently released from hospitals while on neuroleptics or antipsychotics even in the absence of psychotic symptoms or aggressive behavior (3). Although both types of medications (antipsychotics, mood stabilizer agents, and/or anticonvulsants) have proved their effectiveness in the management of mania by reducing the mania scores overall, they do not reduce all manic symptoms with the same intensity. The British Association of Psychopharmacology (BAP) guidelines reports that, in placebo-controlled trials, the atypical antipsychotics used in monotherapy, including aripiprazole, have been shown to be effective in the treatment of acute manic or mixed episodes (4). Factorial approaches to mania have all shown that since there are several clinical subtypes of mania, several clusters of manic symptoms can be identified. Antipsychotic and mood stabilizer agents and/or anticonvulsants do not appear to have equivalent effects on each of these identifiable clusters of symptoms, especially on psychotic features. We think that it is vitally important for future clinical trials conducted in mania treatment to focus on the treatment effects using a factorial approach and an appropriate methodological structure. This question highlights the uncertainty surrounding manic episodes, namely their predominant mood or psychotic nature.

The Europeans consider mania to be more of a mood episode and prefer lithium as first-line treatment, whereas the Americans believe that psychotic symptoms dominate and widely use antipsychotic agents. However, according to the clinical trials currently available, even though antipsychotic agents are certainly effective in reducing the scores on the mania scales, it is not clear whether they can be considered purely as antimanic treatments. Nowadays, there is no clear consensus regarding mania treatment. The question as to whether mood stabilizer agents such as lithium or anticonvulsants (even a combination of both) or antipsychotic agents should preferably be used as first-line treatment of mania remains unanswered and neither the American nor the European guidelines provide an entirely satisfactory answer to this crucial question. Indeed, these two classes can have a somewhat different impact on the underlying symptoms of mania (1). Both therapeutic strategies are feasible and effective (5).

With regard to the American approach (6), three classes of compounds are used as first-line chemotherapy in the management of mania: lithium, sodium divalproate, and antipsychotic agents. In case of severe and purely manic or of mixed episode, the APA advocates the use of a combination of lithium and an antipsychotic or lithium and sodium divalproate. Monotherapy using one of these three compounds (lithium, sodium divalproate, or an antipsychotic) is recommended for less severe episodes. Atypical antipsychotics (olanzapine and risperidone) are preferably used compared to typical antipsychotics due to their better safety profile. Carbamazepine or oxcarbazepine is used only as second-line treatment option. Finally, clozapine is restricted to refractory mania. In case of psychotic mania or combined episodes, the APA recommends the use of antipsychotic treatment. The 2nd edition of the APA guideline for BDs issued in 2010 do not modify its recommendation regarding the treatment of acute mania.

The recommendations for the management of acute mania remain largely unchanged in Canada. Lithium, valproate, and several atypical antipsychotic agents are first-line treatments for acute mania. Monotherapy with asenapine, paliperidone extended release (ER), and divalproex ER have been recently considered as first-line options, as well as adjunctive asenapine (7).

For APA, the severity of the manic episode is the main criterion for treatment decision; on the other hand, for the National Institute for Health and Clinical Excellence (NICE)^1^, the past history of effective antimanic therapy is important while the World Federation of Societies of Biological Psychiatry (WFSBP)^2^ emphasizes the clinical classification of the type of mania as an important criterion.

By contrast, in Europe, lithium remains the reference compound as first-line treatment. There are no clear European consensus recommendations regarding the treatment of mania. Sodium divalproate is usually considered as a second-line treatment in case of lithium contraindication or intolerance. Tohen et al. (8) have carried out a meta-analysis focusing on BD treatments. They have found figures close to 90% of use of typical antipsychotics in the treatment of bipolar patients. These figures are undoubtedly associated with the acute onset of mania and the frequent need to obtain adequate and quick sedation, and, even in some patients, chemical restraint. Sedation is a property of many psychotropic drugs. It can be defined as a decrease in psychomotor and cognitive performances. Many of the classical neuroleptics were extremely sedative and sedation came to be recognized either as adverse or positive (9). In the classification of Delay and Deniker, sleepiness, an extreme sedative effect, is categorized as a side-effect. However, in a more recent classification, the sedative effect is considered to be a major therapeutic effect. Atypical antipsychotics are considerably less sedative than typical antipsychotics while maintaining equivalent or greater antipsychotic activity meaning that sedation is not a prerequisite in antimanic effect (10). Sedation is now increasingly considered as an adverse effect, which should be avoided in manic patients.

The use of typical neuroleptics in the treatment of mania is not devoid of risk and may expose the patient to adverse events. Some authors have pointed out that they could be responsible for exacerbation of the episode and, in the long-term, could adversely affect the disease prognosis (11). Indeed, conventional neuroleptics can trigger mood swings, cause depression, and promote the onset of rapid cycling. The other major risk associated with the use of conventional neuroleptics is the occurrence of acute or delayed extrapyramidal side-effects in manic patients, even more frequently than in schizophrenic patients. These potentially serious side-effects also expose the prescriber to poor patient compliance and can, therefore, have a negative impact upon long-term disease prognosis (12). Finally, conventional neuroleptics can rarely trigger malignant neuroleptic syndrome (13).

Atypical neuroleptics have proven their efficacy in the treatment of mania in several recent studies. They appear to act on different clusters of manic symptoms including psychotic symptoms as well as hyperactivity, agitation, speech, and thought disorders (12). They provide some advantages as compared to typical neuroleptics such as a lower risk of triggering mood swings, an improvement of depressive symptoms, and a better tolerance as compared to conventional neuroleptics, especially as regard to neurological symptoms (14). They can also be used in the treatment of subtypes of mania that have generally a poor response to mood stabilizer agents such as rapid cycling or mixed states. Unlike the situation in the European Union, these drugs can be prescribed as first-line therapy in the USA where they can be used as monotherapy in moderate forms or prescribed concomitantly with lithium or divalproate in more severe or even mixed episodes.

A meta-analysis including 68 randomized controlled trials systematically reviewed from 1980 to 2010 (16,073 participants) has compared any of the following pharmacological drugs at therapeutic dose range for the treatment of acute mania in adults: aripiprazole, asenapine, carbamazepine, valproate, gabapentin, haloperidol, lamotrigine, lithium, olanzapine, quetiapine, risperidone, topiramate, and ziprasidone (15). The main outcomes were the mean changes of the mania rating scales and the number of patients who dropped out of the allocated treatment at 3 weeks. An intention-to-treat analysis was performed. Overall, antipsychotic drugs were significantly more effective than mood stabilizers. Risperidone, olanzapine, and haloperidol should be considered as the best options for the treatment of manic episodes. This meta-analysis is in agreement with a previous one (16) focusing mainly on second-generation antipsychotic monotherapy in acute mania but in this latter study there was no comparison with classical antipsychotics. Aripiprazole has shown higher levels of response and tolerability (less sedative) as compared to haloperidol in the treatment of acute manic episodes. At week 12, significantly more patients taking aripiprazole (49.7%) were in response and receiving therapy compared with those taking haloperidol (28.4%; *P* < 0.001). Continuation rates differed markedly between treatments (week 12: aripiprazole, 50.9%; haloperidol, 29.1%). Extrapyramidal adverse events were more frequent with haloperidol than aripiprazole (62.7 vs. 24.0%) (17).

## Conclusion

There is still no clear consensus regarding the treatment of mania. Although the North Americans have established and regularly updated guidelines related to the management of mania, the same cannot be said in Europe.

Excluding treatments that have not proved effectiveness in methodologically validated trials, two classes of treatments have shown effectiveness: mood stabilizer agents and/or anticonvulsants (such as lithium, divalproate, or carbamazepine) and antipsychotics (typicals or atypicals). Among all antipsychotics, aripiprazole seems the most interesting compound although its use leads to gastrointestinal disturbances and movement disorders. Comparative trials using other compounds than haloperidol and lithium are scarce, drawing conclusions about the use of aripiprazole as a first-line or second-line monotherapy treatment would be premature (18).

In the USA, lithium, divalproate, or antipsychotics can be used as first choice treatment. In Europe, lithium is still considered as first-line therapy whilst divalproate and atypical antipsychotics are used only as second-line treatment. Conventional antipsychotics, which should no longer be recommended in the treatment of mania, given their low safety profile, are still widely used in Europe.

However, antipsychotic and mood stabilizer agents do not seem to have the same effects on each of the clusters of manic symptoms, especially on mood or psychotic clusters. In the future, clinical trials may focus on this important question using an appropriate methodological design. However, from the standpoint of the clinical trials currently available, even though antipsychotic agents are certainly effective in reducing the overall scores on the mania scales, whether they can be considered real antimanic treatment remains an unanswered question.

Even if all atypical antipsychotics do appear to have a potential role in the treatment of BD, especially during manic episodes, it would be erroneous to consider all antipsychotics as real antimanic drugs (11). A “gold standard” antimanic therapy would effectively manage all sorts of manic symptoms while having a good safety profile. Therefore to date for the authors, the atypical antipsychotics of the “third generation” as aripiprazole which is a partial agonist at the dopaminergic receptor level (19) and very easy to use depending on the wide large of posology, seems to be a real antimanic drug even if there is no difference with lithium in the controlled clinical trials (18). Aripiprazole therefore, is a significant player in the current portfolio of antimanic pharmacological treatments (20).

## Conflict of Interest Statement

The authors declare that the research was conducted in the absence of any commercial or financial relationships that could be construed as a potential conflict of interest.

## Footnotes

^1^http://guidance.nice.org.uk/

^2^http://www.wfsbp.org/activities/wfsbp-treatment-guidelines.html
